# Associations Between Skeletal Muscle Mass, Grip Strength, and Physical and Cognitive Functions in Elderly Women: Effect of Exercise with Resistive Theraband

**DOI:** 10.20463/jenb.2019.0023

**Published:** 2019-09-30

**Authors:** Insu Kwon, Ji-Seok Kim, Chul-Ho Shin, Yoonjung Park, Jong-Hee Kim

**Affiliations:** 1. Department of Physical Education, Hanyang University, Seoul Republic of Korea; 2. Department of Sports and Health Care, Namseoul University, Cheonan Republic of Korea; 3. Department of Health and Human Performance, University of Houston, Texas USA

**Keywords:** Theraband exercise, Body composition, Grip strength, Physical function, Elderly women

## Abstract

**[Purpose]:**

The purpose of this study was to identify the relationships between muscle mass, muscle strength, and physical and cognitive functions and to examine the effects of resistive Theraband® exercise on sarcopenia-associated variables in the older population.

**[Methods]:**

A total of 28 elderly women (age: 69.90 ± 0.8 years) participated in this study, 15 of whom underwent elastic band exercise for 1 hour per day, twice per week for 8 weeks. The correlation analysis was conducted to identify the associations between body composition, skeletal muscle mass indices, grip strength, and physical and cognitive functions. All variables were assessed at baseline and post-exercise.

**[Results]:**

Skeletal muscle mass was significantly associated with grip strength and physical function. Gait speed was positively correlated with grip strength and physical function, but not with cognitive function. Theraband® exercise significantly improved gait speed and physical function.

**[Conclusion]:**

The present data suggest that skeletal muscle mass is highly correlated with grip strength and physical function. Eight weeks of resistive Theraband® exercise favorably affects sarcopenia by improving gait speed and mobility of elderly women.

## INTRODUCTION

The rapid growth of the elderly population has become a major public concern. In South Korea, individuals aged 65 and over accounted for 14.3% of the population in 2018, and this proportion is expected to reach 40.1% in 2060^1^. These changes have led to older people paying more attention to health care and the management of age-associated health problems such as sarcopenia^2^. Sarcopenia is a geriatric syndrome defined as age-induced progressive loss of skeletal muscle mass accompanied by a decline in muscle strength and physical function^2^^,^^3^. Because skeletal muscle wasting with age is a major risk factor for frailty, fall, disability, morbidity, and mortality, developing effective prevention and treatment for older adults is necessary.

Resistance exercise has been suggested as one of the most effective interventions to prevent and attenuate sarcopenia^4^. The American College of Sports Medicine recommended resistance exercise as a powerful modality for increasing the muscle mass and strength for the elderly^5^. In a meta-analysis of 49 studies, Peterson et al.^6^ reported that resistance exercise effectively reinforced the elderly’s muscle strength. Elastic Theraband® has been used to provide a resistive type of exercise for major muscle groups with isotonic and isometric muscle contractions. Exercise with Theraband® generates a constant and controlled resistive force. Theraband® exercise also provides practical benefits, because the exercise can be conducted almost everywhere and can be easily modified to fit the patients’ specific needs. Falhman et al.^7^ demonstrated that resistance exercise with an elastic Theraband® could increase muscle mass and strength in elderly individuals with functional limitations. Treiber et al.^8^ demonstrated that Theraband® exercise could be beneficial for improving functional performance in athletes.

Skeletal muscle mass, grip strength (muscle strength), and gait speed (physical function) are major indicators for determining sarcopenia. Many studies have demonstrated positive relationships between skeletal muscle mass, muscle strength, and physical function, while a variable degree of positive correlations or independent associations between these measures have been also reported^9^^,^^10^. For the elderly, lower cognitive function is linked with a greater risk of physical dysfunction and disability^11^. Although age-associated neuronal dysfunction may lead to a decline in muscle mass and physical function, the relationships between muscle mass, muscle strength, and physical and cognitive functions are not clear. In addition, the beneficial effects of resistance exercise on muscle mass and function in older adults are well established; however, studies evaluating the effects of resistance exercise with a Theraband® on elderly women are very limited. Therefore, the purpose of this study was to identify the relationships between muscle mass, muscle strength, and physical and cognitive functions and examine the effect of resistance exercise on sarcopenia-associated variables in older women.

## METHODS

### Subjects

A total of 28 elderly women (69.9±0.8 years old) were recruited from a community welfare center (Table 1). Each subject agreed to participate in the study and signed an informed consent form prior to its initiation. All procedures were approved by the University’s Institutional Review Board (HYI-17-096-1). Subjects who had no difficulty performing physical tasks and no previous medical diagnosis were included in the study. 

**Table 1.** General characteristics of all subjects (N = 28)

Variables	mean ± SEM
Age (years) Height (cm) Body weight (kg) BMI (kg/m^2^)	69.90 ± 0.80 152.73 ± 0.94 55.46 ± 1.14 23.71 ± 0.47
SMI (ASM/body weight x100) ASM (Appendicular skeletal muscle mass, kg) ASM/Height^2^ (kg/m^2^)	25.98 ± 0.49 14.37 ± 0.33 6.15 ± 0.11
K-MMSE (score)	25.04 ± 0.50
Body fat percentage (%) Fat mass (kg) Left upper limb muscle mass (kg) Right upper limb muscle mass (kg) Trunk muscle mass (kg) Left lower limb muscle mass (kg) Right lower limb muscle mass (kg)	32.25 ± 1.09 18.06 ± 0.88 1.82 ± 0.05 1.84 ± 0.05 16.75 ± 0.30 5.36 ± 0.13 5.36 ± 0.13
Balance test (sec) Chair stand test (sec) Grip strength (kg) Gait test (sec) Gait speed (m/sec) TUG test (sec)	4.00 ± 0.00 9.75 ± 0.45 18.94 ± 0.86 3.38 ± 0.14 1.24 ± 0.05 9.05 ± 0.31

Values are mean ± SEM.

BMI (body mass index), SMI (skeletal muscle mass index), K-MMSE (Korean version of Mini Mental State Examination), TUG (Timed Up and Go)

### Exercise program 

Fifteen subjects conducted Theraband® exercise. The exercise program was provided to subjects twice a week for 8 weeks. The program consisted of a warming up exercise for 10 min, followed by the main exercise for 40 min, and a cool down exercise for 10 min. The neck, shoulder girdle, elbow joint, wrist, trunk, hip joint, knee joint, and ankle joint were mainly exercised with yellow and green colored elastic bands. In this regard, the Theraband® was color-coded in the following order of increasing resistance: tan, yellow, red, green, blue, black, silver and gold. Each subject began the program using the yellow Theraband®. As the subjects performed the ability to complete 15 repetitions without difficulty, we progressed to using the stronger, green-colored Theraband®. Taking into account the physical conditions of each subject, exercise intensity was gradually increased every 2 weeks by adjusting exercise tempo, repetition (6–12 times), and number of sets (3–5 sets).

### Outcome Measures

Body composition was measured using a bioelectrical impedance analysis device (BIA, Inbody 720, Biospace, Korea). Skeletal muscle mass index (SMI) which is weight (kg)-adjusted appendicular skeletal muscle mass (ASM) as well as ASM/height^2^ which is height square (m^2^)-adjusted ASM were calculated for sarcopenia indices^12^^,^^13^. The grip strength was measured in both hands using a digital hand dynamometer (Smedley, Takei. Japan). Gait speed was determined by the Short Physical Performance Battery (SPPB) test. The SPPB test, which comprises a balance test, chair stand test, and gait test, was also conducted to evaluate physical function^14^. The Timed Up and Go (TUG) test was performed to assess mobility and physical function^15^. Cognitive functions were determined by the Korean version of the Mini Mental State Examination (K-MMSE) test^16^.

### Statistical analysis

Pearson’s correlation analysis was used to identify the association between measured variables. To determine the mean difference of each variable before and after exercise, we used the paired sample t-test. SPSS software program (Version 21.0 for Windows, IBM Corp., Armonk, NY, USA) was used for all analyses. The effect size was measured as Cohen’s d. All values are presented as mean ± SEM, and statistical significance was set at *p* < 0.05.

## RESULTS

### Relationships between Muscle Mass, Grip Strength, and Physical and Cognitive Functions 

**Figure 1.**
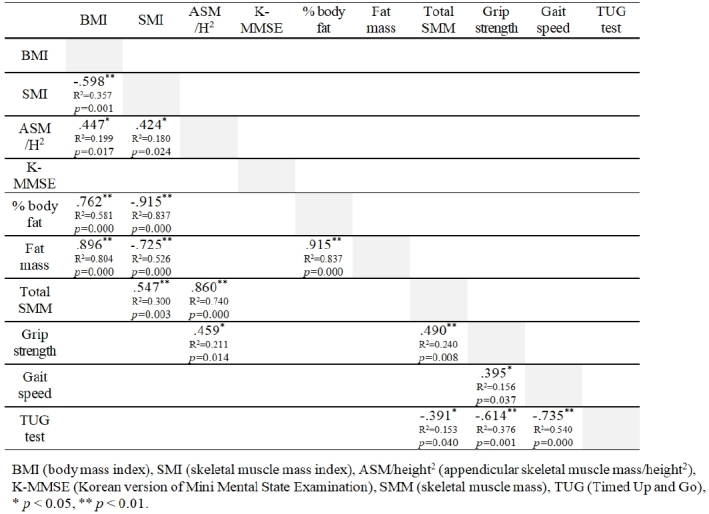
Correlations among body composition, grip strength, and physical and cognitive functions in all subjects (N = 28)

Figure 1 shows the results of correlation analysis among muscle mass, grip strength, and physical and cognitive functions. SMI showed a positive correlation with skeletal muscle mass (*r* = 0.547, *p* = 0.003) and ASM/height^2^ (*r* = 424, *p* = 0.024) and a negative correlation with body fat percentage (*r* = -0.915, *p* < 0.001) and body fat mass (*r* = -0.725, *p* < 0.001). Skeletal muscle mass was significantly associated with grip strength (*r* = 0.490, *p* = 0.008) and physical function in the TUG test (*r* = -0.391, *p* = 0.040), but not with gait speed. Instead, gait speed was better associated with grip strength (*r* = 0.395, *p* = 0.037) and physical function in the TUG test (*r* = -0.735, *p* < 0.001). There were no direct relationships between cognitive function for K-MMSE and SMI, ASM/height^2^, muscle mass, grip strength, gait speed, or physical function. 

### Effect of Theraband® Exercise on Body Composition, Grip Strength, Gait Speed, and Physical and Cognitive Functions

The change of body composition before and after Theraband® exercise is shown in Table 2. There was no difference in body weight, BMI, body fat percentage, body fat mass, and muscle mass between baseline and post-exercise. Eight weeks of Theraband® exercise significantly increased gait speed (*p* = 0.003) and integrated physical functions (*p* = 0.010); however, exercise did not change SMI or grip strength. Cognitive function after exercise exhibited an increased trend (*p* = 0.106) that was not statistically different compared with the baseline.

**Table 2.** Changes in each variable between subjects at baseline and post-exercise with Theraband® (N = 15)

Variables	Baseline	Post-exercise	*p*	Effect size
Body weight(kg) BMI(kg/m^2^)	56.48±1.71 23.67±0.66	56.15±1.75 23.56±0.69	0.227 0.574	0.33 0.15
SMI(ASM/Body weight x100) ASM(kg) ASM/Height2(kg/m^2^)	26.21±0.77 14.65±0.47 6.17±0.16	26.21±0.72 14.67±0.51 6.18±0.17	0.985 0.870 0.946	0.00 -0.04 -0.02
K-MMSE(score)	25.07±0.67	26.47±0.60	0.106	-0.45
Body fat percentage(%) Fat mass(kg) Left upper muscle mass(kg) Right upper muscle mass(kg) Trunk muscle mass(kg) Left lower muscle mass(kg) Right lower muscle mass(kg)	32.21±1.63 18.44±1.36 1.83±0.07 1.84±0.06 16.89±0.42 5.47±0.18 5.50±0.19	31.42±1.54 17.83±1.27 1.87±0.08 1.85±0.08 16.92±0.49 5.46±0.20 5.49±0.20	0.181 0.089 0.222 0.637 0.816 0.878 0.793	0.36 0.47 -0.33 -0.12 -0.06 0.04 0.07
Balance(sec) Chair stand test(sec) Grip strength(kg) Gait test(sec) Gait speed(m/sec) TUG Test(sec)	4.00±0.00 9.52±0.39 19.53±1.14 3.50±0.20 1.19±0.06 9.04±0.36	4.00±0.00 9.10±0.37 19.86±1.29 3.00±0.11 1.36±0.05 8.28±0.38	1.00 0.274 0.567 0.012* 0.003** 0.010*	- 0.29 -0.15 0.74 -0.91 0.77

Values are mean ± SEM.

BMI (body mass index), SMI (skeletal muscle mass index), ASM (appendicular skeletal muscle mass),

K-MMSE (Korean version of Mini Mental State Examination), TUG (Timed Up and Go)

* *p* < 0.05, ** *p* < 0.01.

## DISCUSSION

The purpose of this study was to identify, for the first time, the effects of Theraband® exercise on skeletal muscle mass, grip strength, and physical and cognitive functions in elderly women. Regarding our data (Figure 1), total skeletal muscle mass showed direct positive correlations with sarcopenia-related parameters (SMI and ASM/height^2^). In addition, total skeletal muscle mass of older adults displayed positive correlations with grip strength and physical function. The results of this study are consistent with previous findings^17^^,^^18^ and indicate that skeletal muscle mass, strength, and function reflect clinical significance of sarcopenia such as loss of independency, risk of fall, and functional impairment. Conversely, some studies have reported independent or no associations between muscle mass, strength, and physical function. Visser et al.^10^ reported no relationship between muscle mass loss and physical dysfunction with age. Park et al.^19^ demonstrated that a higher muscle mass did not represent greater muscle strength in the elderly population. Discrepancies in results between studies may be explained by the different comorbid conditions in the patients, such as obesity, diabetes, asthma, and osteoporosis, as well as individual status of nutrition, physical activity, and other lifestyle factors^20^.

It is well known that exercise training in the elderly improves muscle function, metabolic response, and physical performance^21^^-^^23^; however, research investigating the effect of Theraband® exercise on sarcopenia is limited^24^^,^^25^. Previous studies have reported that Theraband® resistance exercise improved body composition, muscle quality, and physical function in the elderly^26^^,^^27^ and patients with sarcopenic obesity^28^. Hofmann et al.^29^ reported that 6 months of elastic band resistance training increased muscle quality of the lower limb but did not alter muscle quality of the upper limb. The data from the current study shows an improvement of physical function in older women, in agreement with previous findings; however, it is possible that Theraband® exercise affecting muscle mass and/or muscle function may influence existing sarcopenia.

Sarcopenia is defined as a loss of skeletal muscle mass with aging accompanied by a reduction in muscular strength and physical function^30^. To diagnose sarcopenia, three indices including SMI, grip strength, and gait speed were suggested by the Asian Working Group for Sarcopenia (AWGS)^31^. Kim et al.^32^ used the height-adjusted appendicular skeletal muscle mass (ASM/height^2^) to determine the prevalence of sarcopenia in the Korean population. When applying the muscle mass for sarcopenia and the cutoffs from his study, five subjects were pre-sarcopenic (class 1 sarcopenia) before Theraband® exercise and four subjects had class 1 sarcopenia after exercise (data was not shown). In addition, when the criteria from the AWGS^33^ were applied, there were five sarcopenic patients (2 with sarcopenia, 3 with severe sarcopenia) before Theraband® exercise, but only two sarcopenia patients (both with severe sarcopenia) after exercise (data was not shown). These results indicate that the Theraband® exercise program may be more effective in preventing and attenuating sarcopenia by improving muscle function rather than by increasing muscle mass.

The data from the current study showed that physical function determined with the TUG test was significantly improved, from 9.04 sec at baseline to 8.28 sec after Theraband® exercise (Table 2). This result may imply that increased gait speed with an enhanced movement coordination causes an improvement of integrated physical performance^34^. Our results also showed that there was no change in SPPB, likely due to the subjects being in a normal condition. This may be associated with an ability to keep health status effectively throughout the Theraband® exercise training periods. These results are in line with the data from a 1-year mixed strength training program^35^, which used the same equipment to train elderly females. Song et al.^6^ reported balance exercise in combination with Theraband® use improves stability, trunk proprioception, and postural control. Therefore, the results of this study demonstrate that Theraband® exercise may be an effective intervention for improving integrated physical performance in elderly women.

Some studies have shown that sarcopenia and physical dysfunction with age are significantly associated with cognitive impairment^36^^,^^37^, but others demonstrated no relationship^38^^,^^39^. In the current study, we found that cognitive function was not related to any testing variables in older women. In addition, protection against age-associated sarcopenia and physical dysfunction through Theraband® exercise did not appear to be associated with cognitive function. Although further studies are needed to verify the relationship between sarcopenia and physical and cognitive function in the elderly women, this finding is partly supported by the data from a recent study where only men showed a significant association between physical and cognitive function^40^. In addition, different approaches used to assess sarcopenia and physical and cognitive functions among studies might play a role in the divergent results. Finally, further studies are warranted to study the timeline response between exercise-induced attenuation of cognitive dysfunction and reversal of age-related reduction in motor neuron-muscle fiber interaction and re-innervation in the elderly.

Limitations of this study are related to the short-term training periods used and low training intensity, which might explain the lack of significant changes in muscle mass and grip strength. Older adults may need to perform higher intensity and volume of exercise to stimulate muscle protein synthesis, but the challenge lies in setting the resistive force relative to the one repetition maximum that produces muscle hypertrophy^41^. In addition, we could not completely control the nutritional status and daily habits of subjects in this study. 

The present exercise protocol exhibited a significant improvement in gait speed and physical function after 8 weeks of Theraband® exercise in elderly women, indicating that this type of exercise may have beneficial effects on the prevention and attenuation of age-associated sarcopenia.
